# A comprehensive transcriptomic landscape of cholangiocarcinoma based on bioinformatics analysis from large cohort of patients

**DOI:** 10.1038/s41598-021-93250-4

**Published:** 2021-07-01

**Authors:** Hongguang Li, Lingxin Qu, Haibin Zhang, Jun Liu, Xiaolu Zhang

**Affiliations:** 1 Department of Hepatobiliary Surgery, Shandong Provincial Hospital, Cheeloo College of Medicine, Shandong University, Jinan, 250021 Shandong China; 2 Department of Hepatobiliary Surgery, Shandong Provincial Hospital Affiliated to Shandong First Medical University, Jinan, 250021 Shandong China; 3 Department of Physiology and Pathophysiology, School of Basic Medical Sciences, Cheeloo College of Medicine, Shandong University, Jinan, 250012 Shandong China

**Keywords:** Cancer, Molecular biology, Gastroenterology

## Abstract

Cholangiocarcinoma (CCA) is a group of malignancies emerging in the biliary tree and is associated with a poor patient prognosis. Although the anatomical location is the only worldwide accepted classification basis, it still has bias. The current study integrates the whole-genome expression data from several big cohorts in the literature, to screen and provide a comprehensive bioinformatic analysis, in order to better classify molecular subtypes and explore an underlying cluster mechanism related to anatomy and geographical regions. Differentially expressed protein-coding genes (DEGs) were identified for CCA as well as subtypes. Biological function enrichment analysis—Kyoto Encyclopedia of Genes and Genomes pathway enrichment analysis—was applied and identified different DEGs enriched signaling pathways in CCA subtypes. A co-expression network was presented by Weighted gene co-expression network analysis package and modules related to specific phenotypes were identified. Combined with DEGs, hub genes in the given module were demonstrated through protein–protein interaction network analysis. Finally, DEGs which significantly related to patient overall survival and disease-free survival time were selected, including *ARHGAP21*, *SCP2*, *UBIAD1*, *TJP2*, *RAP1A* and *HDAC9*.

## Introduction

The human biliary tree, also known as the biliary tract or biliary system is a series of ductular tissues responsible for the drainage of bile produced by the liver and pancreatic secretions from the pancreas^1^. The biliary tree can be subdivided into intrahepatic and extrahepatic parts. The intrahepatic biliary tree starts at the level of canals of Hering, expanding to bile ductules and interlobular bile ducts. Interlobular bile ducts continue into septal, area and segmental bile ducts^1,2^. Based on their size, interlobular and septal bile ducts are considered as small intrahepatic bile ducts (< 300 μm in diameter); whereas area and segmental are considered as large intrahepatic bile ducts (> 300 μm in diameter). Small intrahepatic ducts are lined with small and cuboidal-shaped cholangiocytes while the surface epithelium of large ducts is composed of tall and cylindric cholangiocytes and variably contains mucin-producing cells. The extrahepatic biliary tree comprises the right and left hepatic ducts, the common hepatic duct, the bile duct (i.e. choledochus), the cyctic duct and the gallbladder^3^.

**Figure 1:**
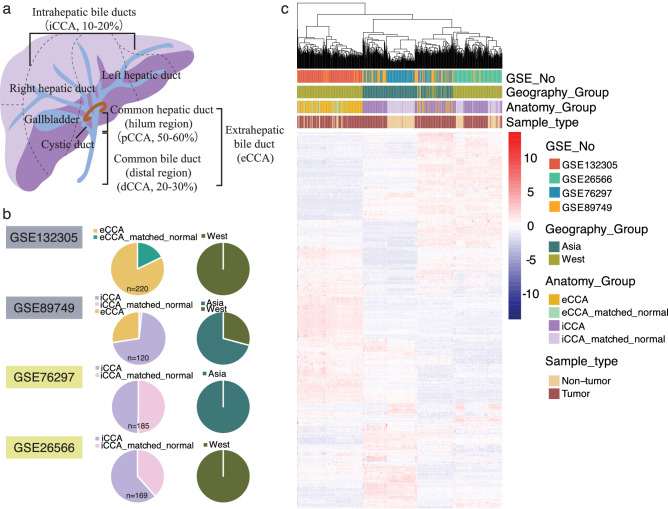
An overview of all datasets used in the current study and clustering. (**a**) An overview of the biliary tree and three subtypes of CCA according to anatomical location. (**b**) Illustration of the four GEO databases. (**c**) A heatmap clustering based on 3000 genes randomly chosen by the software. Rows represent genes, and columns represent samples.

Cholangiocarcinoma (CCA) constitutes a genetically biologically heterogenous group of malignancies emerging in the biliary tree^4,5^. It can arise from epithelial cells in the biliary surface epithelium (i.e. cholangiocytes) and in peribiliary glands, and possibly also from progenitor cells or even mature hepatocytes. CCA can be classified into intrahepatic (iCCA), perihilar (pCCA), and distal cholangiocarcinoma (dCCA) according to their anatomical site of origin^6^. pCCA and dCCA can also be collectively referred to as ‘extrahepatic’ (eCCA)^7^ (Fig. 1a). Although these three CCA subtypes have common features, they also have important inter- and intra- tumor differences that can affect the pathogenesis and outcomes^8–12^. CCA is a rare cancer. The highest rates of CCA are in South East Asia (Northeast Thailand, Cambodia, and Laos), where the incidence of infection by liver flukes such as *Opisthorchis viverrini* and *Clonorchis sinensis* is high. CCA is relatively rare in Western countries where risk factors such as primary sclerosing cholangitis, hepatolithiasis and choledochal cysts predominate. CCA mortality is higher in countries/regions in Asia versus those in the West. Variations in incidence probably reflect differences in local risk factors and potential genetic predispositions^3^.

Actually, the classification by anatomy is biased by some pitfalls. Firstly, there are no differences between pCCA and iCCA that originate from large bile ducts; secondly, CCA is frequently diagnosed at an advanced stage especially for pCCA, where distinguishing between an intra-hepatic or extra-hepatic location proves challenging^13^. On the other hand, due to strong heterogeneity, the current understanding of the molecular mechanisms of CCA is still not comprehensive. Studying the pathogenesis of CCA and identifying hub genes that are involved in the development of CCA remain a major challenge.

Most of the studies on CCA showed the molecular clusters are not simply recapitulating anatomical variation, but as each of them either included a single subtype (iCCA or eCCA), or the patient number of subtypes was not comparable, it hinders a comprehensively molecular landscape of CCA subtyping. Here we integrate the whole-genome expression data from several big cohorts (GSE132305^14^, GSE89749^15^, GSE76297^16^, GSE26566^17^) in the literature, to screen and provide a comprehensive bioinformatic analysis, in order to better classify molecular subtypes and explore an underlying cluster system related to anatomy, geographical regions, and et al. The present study aimed to explore the biological functions, signaling pathways and potential prognostic biomarkers differences through transcriptomic analysis involved in CCA with different anatomical locations.

## Results

### Characteristics of the four GSE databases involved in the current study

As shown in Fig. 1b, four GSE databases were included in this study. GSE132305 contains exclusively eCCA cases and matched noncancerous tissues, all from western countries^14^. GSE89749 includes both iCCA and eCCA patients, from either eastern or western countries^15^. GSE76297^16^ and GSE26566^17^ involve exclusively iCCA/matched noncancerous tissues, but cases in GSE76297 from Asia and cases in GSE26566 from western countries. In summary, 694 samples totally were collected in the current study for further analysis, including 283 iCCA cases and 158 matched noncancerous tissue samples, as well as 215 eCCA cases and 38 matched noncancerous tissue samples. Out of those samples, 405 from West and 289 from Asia.

After elimination of batch effects, extraction of common genes and merging of the four cohorts, in total 14,552 genes were identified to be commonly detected by the four platforms. Further analysis was conducted based on this list of genes.

### Hierarchical and principal component analysis unsupervised clustering identified distinct subtypes dependent on anatomy and geography

**Figure 2:**
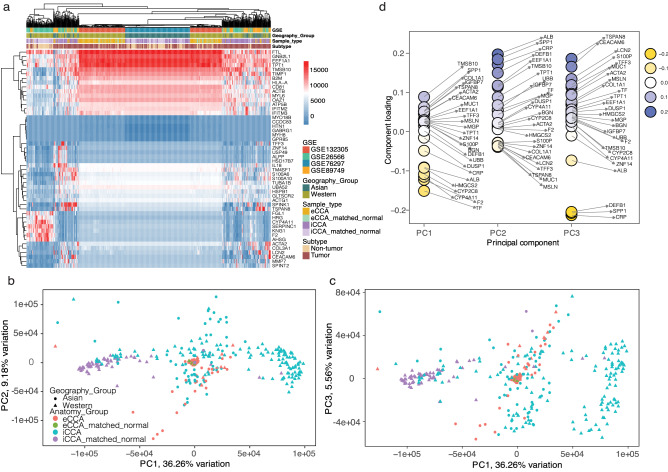
Clustering by pretty heatmaps and PCA. (**a**) A heatmap hierarchical clustering based on the top 40 DEGs. (**b**) The 2D coordinates of each sample profile based on the scores of the first two PCs—PC1 and PC2 in PCA analysis. (**c**) The 2D coordinates of each sample profile based on the scores of PC1 and PC3. (**d**) Contributors of each PC.

A representative gene expression heatmap of 3000 genes randomly chosen by the software was produced following bioinformatics analysis. The hierarchical clustering was based on four factors: batch, geography, anatomy and sample type (either tumor or not). As shown in Fig. 1c, first, the samples were clustered independent of the sample types or batch effects, as tumor samples were randomly mixed with noncancerous tissue samples. Second, among the four factors, anatomy played as a leading role in clustering and all samples were classified into two groups based on it: intrahepatic samples, including iCCAs and matched noncancerous tissues, and extrahepatic samples, including eCCAs as well as matched noncancerous tissues, which was as expected. Third, besides the influences of anatomy, geography or epidemiology played an adjuvant role for clustering. If considering both anatomy and geography, all samples could be classified into three well-distinguished groups: western eCCA cases, Asian iCCA cases and western iCCA cases. Moreover, western eCCA and iCCA groups showed the biggest differences among the three groups. The fourth and the most interesting finding here was that a small sub-group was identified, which included both eCCA and iCCA, from either West or East and accounted for approximately one fifth of all samples. It was hypothesized here that the iCCA cases in this sub-group originated from large intrahepatic bile ducts, which showed similar molecular behaviors as eCCA, and patients in this small group shared similar oncogenic factors independent of the geography locations. We next repeated the hierarchical clustering based on the top 40 DEGs and the heatmap showed similar findings, revealing the classification of CCA patients was highly dependent on tumor anatomical locations and geographical regions (Fig. 2a).

Next, principal component analysis (PCA) was performed (Fig. 2b–d). PCA can simplify the complexity in high-dimensional data while retaining trends and patterns^18^. High-dimensional data are very common in biology like here for CCA, where expression of many genes is measured for each sample. All cases displayed distinct group-bias clustering and individual differences by PCA. When displayed by the scores of the first two PCs—PC1 and PC2 (Fig. 2b), all iCCA and matched noncancerous samples clustered together based on PC2, but extended along the PC1 coordinate showing individual variations. On the contrary, eCCA and matched noncancerous samples were clustered more closely than iCCA based on PC1, but expanded a bit along PC2 factors. All samples exhibited more scatteredly on PC1 and PC3 plot, especially iCCA and matched noncancerous samples, which extending along both PC1 and PC3 (Fig. 2c). All contributors for each PC were shown on Fig. 2d. It didn’t show a clear clustering pattern of CCA by epidemiology on PCA plots.

### WGCNA analysis revealed a specific module highly associated with tumor- and subtype- phenotypes

**Figure 3:**
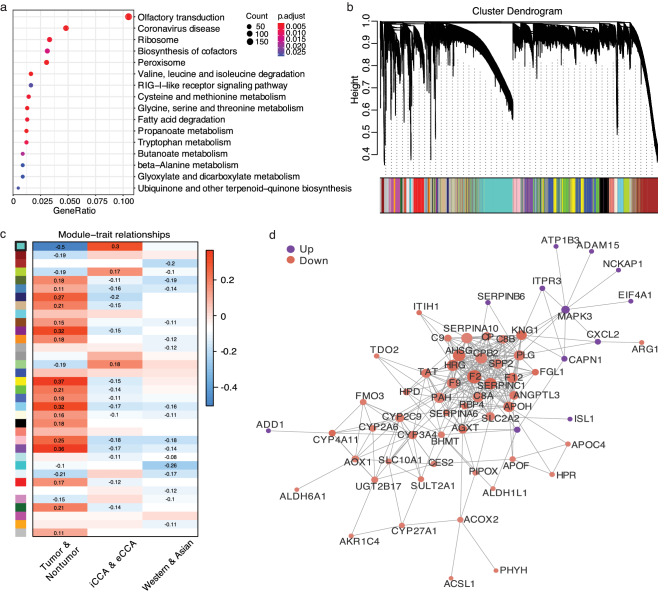
Network construction and module detection. (**a**) Functional enrichment analysis of DEGs by KEGG. The y-axis represents KEGG-enriched terms. The x-axis indicates the fold of enrichment. The size of each dot indicates the number of genes under a specific term. The color of the dots represents the adjusted *p *value. (**b**) Clustering dendrogram of genes with dissimilarity based on the topological overlap, together with assigned module colors using WGCNA analysis. (**c**) Illustration of association between modules and traits in WGCNA. Each row refers to a module eigengene, column to a trait. Module ‘turquoise’ is highlighted with black rectangle with the highest association coefficient. (**d**) PPI network of the DEGs within the selected module. The nodes represent each gene with edges mean the interactions. Purple and red means gene upregulation and downregulation, respectively.

DEGs of all CCAs were identified. A total of 3042 DEGs were distinguished, including 227 upregulated genes and 2815 downregulated genes. Biological function enrichment analysis -KEGG pathway enrichment analysis was applied then (Fig. 3a). DEGs were enriched in such as ‘biosynthesis’, ‘valine, leucine and isoleucine degradation’ and ‘fatty acid degradation’ pathways.

Then a co-expression network was presented by the WGCNA package^19^ (Fig. 3b). Gene modules related to ‘tumor’, ‘subtype’ and ‘geography’ were constructed. 1282 genes included in the module ‘turquoise’ generated in WGCNA were screened as candidates of tumor- and subtype- associated genes (Fig. 3c). Out of this module, 579 DEGs were extracted and hub genes were identified through PPI network analysis. The PPI network suggested that *RBP4*^20^, *SPP2*^21^, *MAPK3*^22^ and another around 30 genes were hub genes (Fig. 3d). However, we could not identify any significant modules/clusters that could indicate any relationship with epidemiology, suggesting that multiple factors like etiology, ethics, heredity and others might interact as geographical impacts.

### DEGs specific to iCCA and eCCA were identified

**Figure 4:**
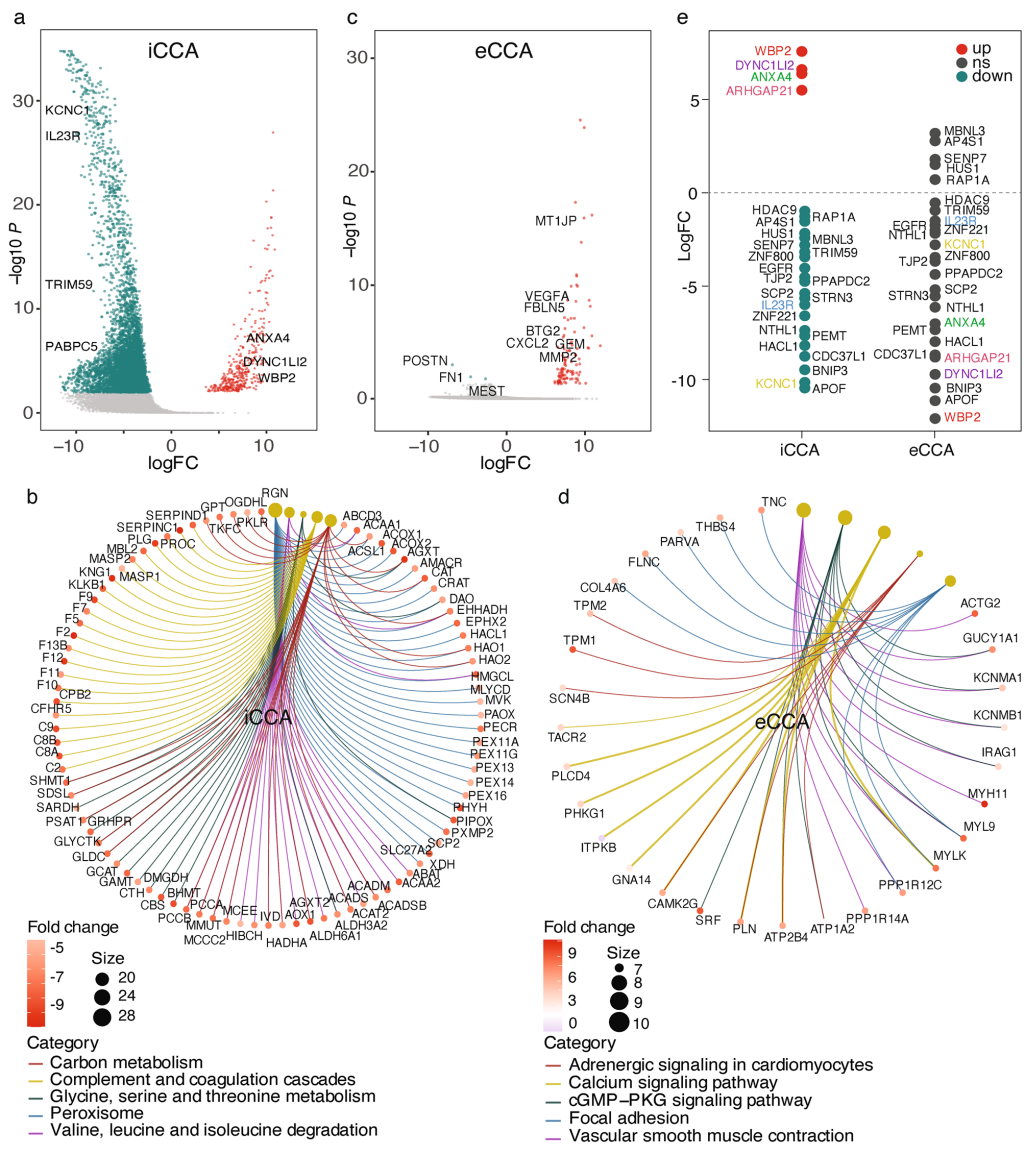
Volcano plots showing significant DEGs in iCCA (**a**) and in eCCA (**b**). The red spots represent significantly upregulated genes, and the green spots represent significantly downregulated genes. (**c**) Expression variations of selected DEGs in iCCA and eCCA. KEGG biological function enrichment analysis of DEGs for iCCA (**d**) and eCCA (**e**) respectively.

To deeply explore the distinctions between iCCA and eCCA, we extracted and analyzed the iCCA samples and eCCA samples respectively, identifying the subtype specific DEGs, as well as their enrichments of biological function. For iCCA, 395 upregulated and 5889 downregulated DEGs were identified (Fig. 4a), KEGG analysis revealed that the hub DEGs were involved mainly in ‘carbon metabolism’, ‘complement and coagulation cascades’ and ‘glycine, serine and threonine metabolism’ pathway (Fig. 4b). Liver is an organ involved in most of the metabolism process of our body, for instance, protein synthesis and carbohydrates production. In the case of iCCA, the normal hepatocytes were replaced by cancer cells and the normal function of liver was impaired, so it is no doubt that the hub downregulated DEGs were mainly involved in carbon and protein metabolism pathways in iCCAs. Compared to iCCA, there were quite fewer DEGs including 117 upregulated and 3 downregulated ones identified (Fig. 4c), KEGG analysis demonstrated that the hub DEGs involved mainly in ‘calcium signaling pathway’, ‘cGMP-PKG signaling pathway’ and ‘focal adhesion pathway’ (Fig. 4d). The calcium signal is a critical regulator of a variety of cellular processes, many of which intersect with those important in cancer progression, such as proliferation and invasiveness^23^. cGMP-PKG signaling pathway regulates a broad array of physiologic processes, and influences anti-proliferative as well as pro-apoptotic mechanisms in multiple carcinomas^24^. Focal adhesion pathway is involved in regulation of migration of various normal and cancer cells^25^. All of those signaling pathways have been reported to be dysregulated in multiple cancer types. It showed a predominant transcriptomic landscape difference between the two subtypes of CCA, which can be illustrated by the different DEGs and biological function enrichments. We picked up 26 DEGs identified in all CCA cases whose expression was confirmed to be related to CCA patient overall survival (OS) time in the TCGA database, and placed them in a plot to compare their expression levels in iCCA and eCCA respectively, it demonstrated that all those 26 DEGs in CCA were also dysregulated in iCCA, but none of them was found significantly dysregulated in eCCA, although some of the DEGs had opposite expression patterns in the two subtypes (Fig. 4e).

### Survival analysis based on significantly dysregulated hub genes

**Figure 5:**
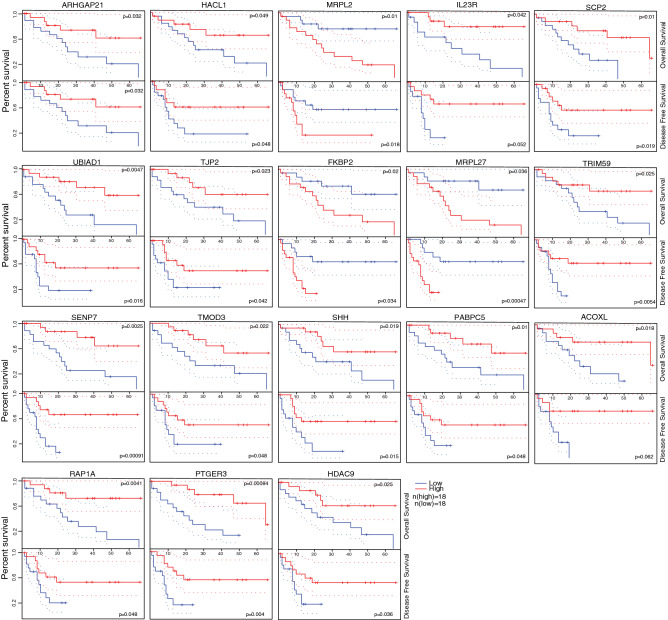
Survival analysis of significantly dysregulated hub genes based on TCGA data. Survival time is recorded in months.

We extracted all DEGs that were shown to be significantly related to both patient OS and disease-free survival (DFS) time in the TCGA database (Fig. 5). For the upregulated DEGs, *ARHGAP21* was the only gene whose expression was found significantly positively related to both patient OS and DFS. ARHGAP21 functions preferentially as a GTPase-activating protein (GAP) for CDC42 and regulates the ARP2/3 complex and F-actin dynamics at the Golgi through control of CDC42 activity^26^. Evidence showed that as a PDZ domain containing protein, ARHGAP21 could interact with the PDZ-binding motif of Claudin-2, in such way promoted breast cancer liver metastasis^27^. It also reported as a down effector of MEC-17, ARHGAP21 was involved in cell spreading, cancer migration and invasion process^28^. Among the downregulated DEGs, the expression of *SCP2*, *UBIAD1*, *TJP2*, *RAP1A*, *HDAC9* et al.were significantly positively related to patient OS and DFS. The expression of *FKBP2*, *MRPL2* and *MRPL27* were negatively related to patient OS and DFS. SCP2 protein is an intracellular lipid transfer protein and involved in lipid metabolism^29^ and bile acid synthesis^30^, SCP2 was reported to be dysregulated in pituitary adenomas^31^. One study indicated that lower expression of SCP2 reflected poor prognosis and survival time of CCA patient^32^. UBIAD1 is a protein involves in cholesterol and phospholipid metabolism^33^. Studies showed UBIAD1 is a target of onco-microRNA miR-4644 in bladder cancer^34^. UBIAD1 could inhibit the proliferation of bladder cancer cells via interaction with H-Ras^35^. TJP2 encodes protein functions as a component of the tight junction barrier in epithelial and endothelial cells and is necessary for proper assembly of tight junctions^36^. CLDN6/TJP2/YAP1 interacting axis was induced in hepatocellular carcinoma (HCC) to enhance tumor lineage plasticity and cellular identity change^37^. RAP1A encodes a member of the Ras family of small GTPase, which can regulate signaling pathways that affect cell proliferation and adhesion, playing a role in tumor malignancy^38^. HDAC9 encodes a protein which has sequence homology to members of the histone deacetylase family, by histone acetylation/deacetylation it can alter chromosome structure and affect transcription factor access to DNA^39^. HDAC9 was transcriptionally upregulated in epithelial-mesenchymal transition (EMT)—induced HCC cells^40^.

**Figure 6:**
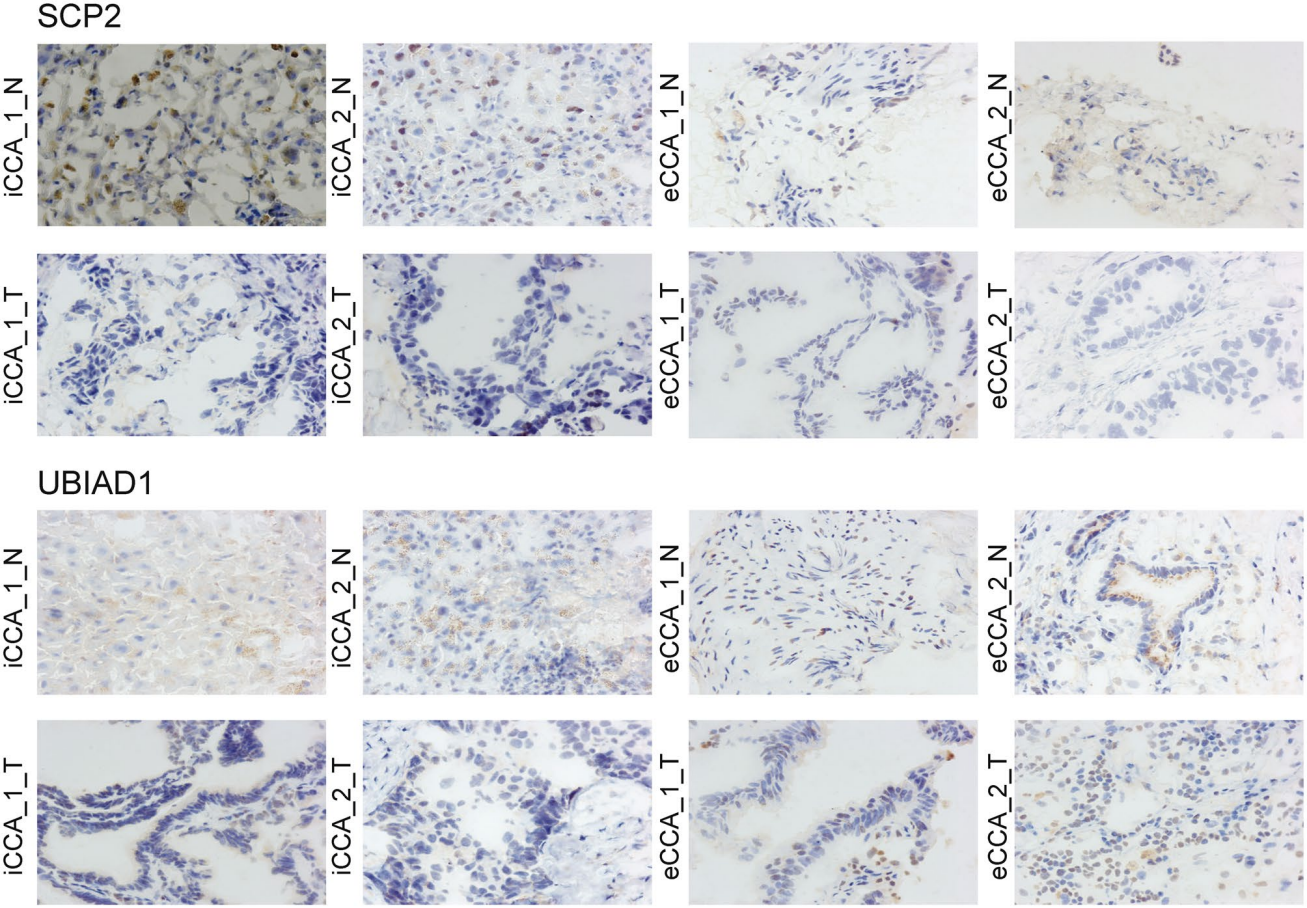
Immunohistochemistry staining of SCP2 and UBIAD1 on 2 iCCA cases and 2 eCCA cases. *N* represents for noncancerous tissue and *T* for tumor specimen.

We performed immunohistochemistry staining of SCP2 and UBIAD1 on 2 iCCA cases and 2 eCCA cases as well as the matched noncancerous tissues. The results showed that both proteins were obviously downregulated in iCCAs, but expression slightly inhibited in eCCA, which was consistent to our bioinformatic findings (Fig. 6). The clinical information of all 4 patients were listed in Table 1.

**Table 1:** Clinical characteristics for enrolled patients.

Patient ID	Gender	Age (year)	Pathology	Tumor grade	TNM staging
iCCA_1	Female	63	T	Moderately differentiated	T1aN1M0, IIIB
iCCA_1	Female	63	N		
iCCA_2	Male	65	T	Poorly differentiated	T1bN0M0, IB
iCCA_2	Male	65	N		
eCCA_1	Male	53	T	Moderately to poorly differentiated	T2N0M0, IIA
eCCA_1	Male	53	N		
eCCA_2	Male	60	T	Highly to moderately differentiated	T3N1M0, IIB
eCCA_2	Male	60	N		

*T* tumor specimen, *N* noncancerous tissue.

## Discussion

It is the only one world widely accepted classification system for CCA based on anatomical locations despite that more and more studies in recent years with high-throughput whole genetics and genome-expression sequencing revealed several molecular subtypes of CCA. In one study, molecular profiling of iCCA has allowed the discovery of 2 distinct transcriptome-based classes: an ‘Inflammation class’ with predominant induction of immune response pathways and less-aggressive clinical behavior, and a ‘Proliferation class’ with chromosome instability and activation of classic oncogenic pathways that correlate with worse outcome^41^. Another recent study conducted on eCCA cohort defined 4 molecular classes of it: ‘Metabolic class’ shows a hepatocyte-like phenotype with activation of the transcription factor HNF4A and enrichment in gene signatures related to bile acid metabolism; ‘Proliferation class’ characterized by enrichment of *MYC* targets, *ERBB2* mutations/amplifications and activation of mTOR signaling; ‘Mesenchymal class’ defined by signatures of epithelial-mesenchymal transition, aberrant TGFβ signaling and poor overall survival; and ‘Immune class’ with a higher lymphocyte infiltration, overexpression of PD-1/PD-L1 and molecular features associated with a better response to immune checkpoint inhibitors^14^. However, due to strong genetic heterogeneity, the current understanding of the molecular mechanisms of CCA is still not comprehensive.

As the classification by anatomy is biased by some pitfalls and most of the studies on CCA in recent years either included a single subtype (iCCA or eCCA), or the patient number of subtypes was not comparable, a better widely accepted molecular classification system of CCA was still not achieved yet. It should be pointed that although previous efforts in the setting of the International Cancer Genome Consortium (ICGC) and TCGA have highlighted the crucial role of anatomical location of CCA, underrepresentation of eCCA and not well seperation of pCCA and dCCA are a big insufficiency. All versions of the main International Classification of Diseases (ICD) have so far failed to include a separate code for the largest group of CCA (pCCA) and previous versions of ICD-Oncology (ICD-O) have cross-referenced pCCA (technically extrahepatic) to iCCA^42^. Importantly, for the first time, subsequenct iterations of both ICD and ICD-O (ICD-11 and ICD-O-4, respectively)—which are due to come into effect in 2021—will have separate codes for recording iCCA, pCCA and dCCA. It is important to ensure the separation of the given anatomical subtypes and to search for distinct subgroups within the subtypes on a molecular and morphological basis in the future research. This goal of a new morpho-molecular classification of CCA can only be reached if clinicopathologically well-characterized cohorts are used.

Genome expression study is a method that is conductive to the application of a systematic comprehensive study of differentially expressed gene interactions and related signaling pathways with high precision. In order to get a more comprehensive understanding of CCA expression profiles, here we combined four large cohorts of CCA patient data and performed multiple bioinformatic analysis methods. First, hierarchical clustering revealed an anatomical position and geography region dependent classification type of all CCA cases, highlighting the importance of tumor cell origin and carcinogenic factors in the pathogenesis of CCA. Second, PCA analysis was conducted in the next step. PCA is an unsupervised learning method, which can find patterns without reference to prior knowledge about whether the samples come from different treatment groups or have phenotypic differences^18^. All CCA cases displayed distinct subtype-bias clustering and individual differences on PCA plots, indicating significant inter-tumor heterogeneity on top of subtype-common features. Third, WGCNA is employed here to detect complex associations between genes and phenotypes. Unlike strategies that rely on DEGs analysis, WGCNA focuses on gene co-expression and correlation networks, which is widely used for biomarkers and therapeutic targets. Genes can be grouped into a co-expression module based on their similar expression profiles^19^. Here with WGCNA analysis, a group of genes, referred to as module or cluster, was identified to be highly related to tumor and subtype phenotype. We further focused on the DEGs in this group of genes and applied PPI network analysis to distinguish hub genes, which are defined as genes with a high degree of connectivity that play an essential role in stabilizing the PPI network structure. Here, some star genes were identified, such as *RBP4*^20^, *SPP2*^21^ and *MAPK3*^22^, those had been confirmed a role in pathological mechanisms. Fourth, DEGs specific to CCA subtypes were explored and compared. It showed that iCCA and eCCA displayed significantly different behaviors at the transcriptome level. It should point out that although thousands of DEGs were identified, a large group of them actually had really low or even none basic expression level, so it makes no sense if the absolute expression changed a bit in cancer but induced a significant fold change. To avoid it, we selected manually and focused on those true DEGs further and discarded the nonsense DEGs. Finally, combined with TCGA data, DEGs that were shown to be significantly related to both patient OS and DFS time were listed. Those genes could serve as biomarkers or therapeutic targets for CCA, but further studies are still needed to confirm those findings.

In conclusion, the findings in the current study with multiple big cohorts of CCA patients could help enhance the current understanding of CCA and provide new insight into distinguishing candidate biomarkers for different subtypes of CCA.

## Methods

### Acquisition of transcriptome data and identification of DEGs

Four Gene Expression Omnibus (GEO) series (GSE)—GSE132305, GSE89749, GSE76297 and GSE26566—were downloaded from the GEO database (http://www.ncbi.nlm.nih.gov/geo/). GSE132305 is based on the platform of Affymetrix Human Genome U219 Array, GSE89749 on Illumina HumanHT-12 V4.0 expression bead chip, GSE76297 used Affymetrix Human Transcriptome Array 2.0 [transcript (gene) version] and GSE26566 is based on Illumina HumanRef-8 v2.0 expression beadchip. Statistical softwareR (version 4.0.3) and packages ‘Limma’ and ‘Tidyverse’ were used for data pre-processing and for performing significance analysis of the DEGs between CCA samples and matched adjacent noncancerous tissues. Genes with an absolute value of log2 fold change (|log2FC|) > 1 and a corrected P-value < 0.0001 were defined as DEGs.

### Clustering analyses

For CCA samples clustering, pretty heatmaps clustering method and PCA analysis^18^ were used. Weighted gene co-expression network analysis (WGCNA)^19^ was used for finding modules/clusters of highly correlated genes and for finding modules related to well-defined phenotypic features. DEGs in the most significant module associated with phenotypic features were identified for further analysis. The protein–protein interaction (PPI) network was built using an online tool STRING (http://string-db.org/) and visualized via Cytoscape software (version 3.7.1; http://cytoscape.org/) to identify the hub genes in the given module.

### Functional and pathway enrichment analyses

Kyoto Encyclopedia of Genes and Genomes (KEGG) is a genome deciphering database and an enrichment analysis tool for the research of biological regulatory networks. Here, KEGG pathway enrichment analysis was applied based on the R package ‘clusterProfiler’. Critical pathways enriched in DEGs were identified. Visualization of KEGG results was conducted by R package ‘ggplot2’. *P* < 0.05 was considered statistically significant for KEGG analysis.

### Survival analysis using GEPIA2

Using GEPIA2 (http://gepia2.cancer-pku.cn/#survival)^43^, Kaplan–Meier survival curves were plotted, and the log-rank test was employed to compare the differences in OS and DFS based on TCGA data with the most differential expression genes identified in the current study.

## Data Availability

All data generated or analyzed during this study are included in this published article. Source data can be referred to https://www.ncbi.nlm.nih.gov/geo/.
